# Infected abdominal aortic aneurysm caused by *Streptococcus pneumoniae* diagnosed using polymerase chain reaction

**DOI:** 10.1186/s40792-015-0085-6

**Published:** 2015-09-16

**Authors:** Kyohei Hatori, Satoshi Ohki, Takao Miki, Hanako Hirai, Kiyomitsu Yasuhara, Tamiyuki Obayashi

**Affiliations:** Department of Cardiovascular Surgery, Isesaki Municipal Hospital, 12-1 Tsunatori-honmachi, Isesaki, Gunma 372-0812 Japan

**Keywords:** Infected abdominal aortic aneurysm, *Streptococcus pneumoniae*, Polymerase chain reaction, Rifampicin-soaked vascular prosthesis

## Abstract

We report the case of a 55-year-old man who initially visited the emergency department of our hospital owing to fever, headache, and neck stiffness. He was diagnosed with meningitis because cerebrospinal fluid culture was positive for *Streptococcus pneumoniae*. After intravenous antibiotic treatment, the patient’s condition returned to normal. On hospital day 20, he complained of lumbar pain with abdominal distension. Because an abdominal computed tomography (CT) scan showed a small sacciform infrarenal abdominal aortic aneurysm, an infected aneurysm was suspected. However, cerebrospinal fluid and blood cultures were negative for *S. pneumoniae*. Seven days later, a second abdominal CT was performed that showed rapid expansion of the sacciform infrarenal abdominal aortic aneurysm. The patient was diagnosed with an infected abdominal aortic aneurysm and underwent surgery for resection of the aneurysm and in situ reconstruction with a rifampicin-soaked vascular prosthesis. Although blood and aneurysmal tissue cultures were negative for *S. pneumoniae*, the autolysin (lytA) gene, which is the target gene of *S. pneumoniae*, was detected in the abdominal aortic wall by using polymerase chain reaction (PCR). Therefore, appropriate molecular diagnostic techniques can be used for the rapid detection of pathogens. An accurate diagnosis can be used to direct postoperative antibiotic therapy.

## Background

Despite recent improvements in surgical techniques and antibiotic therapy, severe aortic infection and a subsequent infected aortic aneurysm remains a lethal condition. Early diagnosis and surgical intervention and prolonged antibiotic therapy are essential for survival [1, 2]. *Streptococcus pneumoniae* aneurysms usually affect the aorta; however, bacterial cultures from blood and aneurysmal tissue may be difficult because of prior antibiotic therapy. We report the case of an infected abdominal aortic aneurysm following pneumococcal meningitis, which was managed by resection and revascularization with rifampicin-soaked vascular prosthesis. Although cultures from the cerebrospinal fluid, blood, and aneurysmal tissue tested negative for *S. pneumoniae*, *S. pneumoniae* DNA was detected from samples taken from the abdominal arterial wall by using polymerase chain reaction (PCR). PCR is a unique technique to identify pathogens, even after antibiotic therapy was initiated.

## Case presentation

**Fig. 1:**
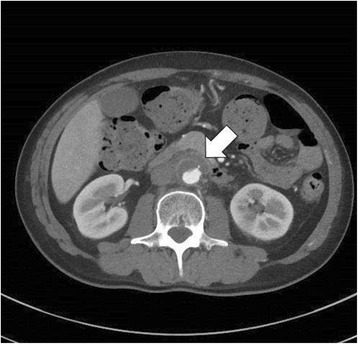
Preoperative CT scan. Axial view showing a small sacciform infrarenal abdominal aortic aneurysm (*white arrow*)

**Fig. 2:**
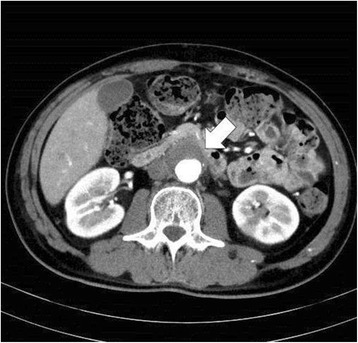
Second preoperative CT scan, seven days after the first CT scan. Axial view showing rapid expansion of the sacciform infrarenal abdominal aortic aneurysm (white arrow)

**Fig. 3:**
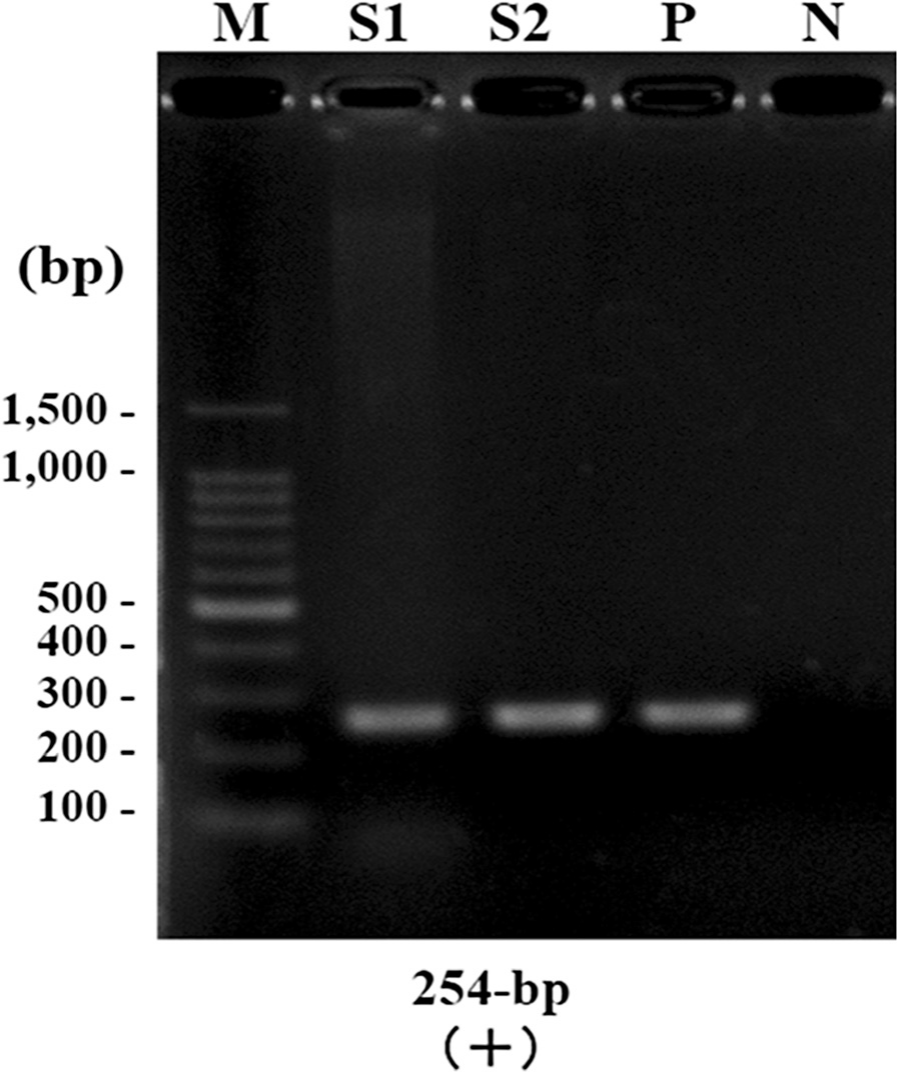
PCR results using primers to the lytA gene. *M* molecular size marker, *S1* DNA sample twofold dilution, *S2* DNA sample fivefold dilution, *P* positive control (LytA gene), *N* negative control

A 55-year-old man initially visited the emergency department of our hospital owing to a 3-day history of fever, headache, mild shoulder pain, and neck stiffness. The patient’s neck stiffness and shoulder pain worsened, and the patient had a depressed level of consciousness. Laboratory data on admission revealed a leukocyte count of 25,600/μl and an elevated C-reactive protein level of 28.3 mg/dl. Cerebrospinal fluid and blood cultures were positive for *S. pneumoniae*. As such, a diagnosis of meningitis was made. After intravenous antibiotic treatment (penicillin G benzathine, 4 million units i.v. every 4 h), the patient’s fever resolved, and his mental status returned to normal. On hospital day 20, he complained of low back pain and abdominal distension. Because an abdominal computed tomography (CT) scan showed a small sacciform infrarenal abdominal aortic aneurysm (Fig. 1), an infected aneurysm was suspected. However, cerebrospinal fluid and blood cultures were negative for *S. pneumoniae*. Transthoracic echocardiography indicated no sign of endocarditis. Laboratory data revealed a leukocyte count of 15,200/μl and a C-reactive protein level of 2.51 mg/dl. Seven days later, a second abdominal CT was performed, which showed rapid expansion of the sacciform infrarenal abdominal aortic aneurysm (Fig. 2). An infected abdominal aortic aneurysm was diagnosed, and the patient underwent surgery for resection of the aneurysm and debridement of tissue. We performed irrigation with copious amounts of saline solution and in situ reconstruction with a rifampicin-soaked bifurcated vascular prosthesis (Triplex 16 × 8 mm, [Terumo Corporation, Tokyo, Japan]). The patient’s omentum was atrophic, so we could not use the tissue for an omental flap around the graft. Pathologic examination of the aorta revealed thrombus formation and a calcified and inflamed aortic wall with neutrophil infiltration. Although cultures from the cerebrospinal fluid, blood, and aneurysmal tissue tested negative for *S. pneumoniae*, the autolysin (lytA) gene, which is the target gene of *S. pneumoniae* [3–6], was detected in abdominal arterial wall samples by using PCR (Fig. 3). PCR analysis accurately diagnosed the infected abdominal aortic aneurysm due to *S. pneumoniae*. Antibiotic agents (meropenem, 0.5 g i.v. every 12 h) were administered intravenously in the hospital for 4 weeks postoperatively until the patient demonstrated normal leukocyte and C-reactive protein levels. Thereafter, oral antibiotic (levofloxacin, 500 mg orally once per day) treatment was continued for 6 months. The patient’s postoperative course was uneventful, and he was discharged on postoperative day 40. No signs of recurrent infection have been observed for 2 years after the operation.

### Discussion

The natural progression of infectious aortitis is the rapid and unusual dilatation of an aneurysm [7]. Therefore, diagnosis must be done early because untreated infectious aortitis is associated with a high rate of aortic rupture and mortality [8]. *Salmonella* and *Staphylococcus aureus* represent the most frequent microbial agents in infectious aortitis [9], while *S. pneumoniae* is a rarer pathogen [10, 11]. *S. pneumoniae*-related aneurysms usually affect the aorta; however, bacterial cultures from blood and aneurysmal tissue may be difficult because of prior antibiotic therapy. Therefore, it is important to combine bacterial cultures with another microbiological method. The lytA gene, which encodes the autolysin protein, is one of the target genes for *S. pneumoniae*. Several studies using specimens of the cerebrospinal fluid, blood, and sputum have reported that the lytA gene constitutes the most sensitive and specific assay in several target genes for *S. pneumoniae* [3–6]. In our case, although cultures from the cerebrospinal fluid, blood, and aneurysmal tissue tested negative, lytA gene was detected using PCR in samples obtained from the abdominal arterial wall. Infected abdominal aortic aneurysm due to *S. pneumoniae* was accurately diagnosed. Dickinson et al. [12] reported that they made a diagnosis of multiple peripheral pneumococcal mycotic aneurysms by using the PCR technique. On the other hand, our case is the first to report that the lytA gene was detected in abdominal arterial wall. Our data suggest that appropriate molecular diagnostic techniques such as PCR can be used for the rapid detection of pathogens. Rapid and accurate diagnosis can be used to direct antibiotic therapy immediately after operations.

## Conclusions

In conclusion, we report a case of infected abdominal aortic aneurysm following pneumococcal meningitis. Although cultures from the cerebrospinal fluid, blood, and aneurysmal tissue tested negative for *S. pneumoniae*, the lytA gene was detected using PCR in samples obtained from the abdominal arterial wall. Appropriate molecular diagnostic techniques can be used for the rapid detection of pathogens. An accurate diagnosis can be used to direct postoperative antibiotic therapy.

## Consent

Written informed consent was obtained from the patient for publication of this case report and any accompanying images. A copy of the written consent is available for review by the Editor-in-Chief of this journal.

## Abbreviations

CT: Computed tomography
DNA: Deoxyribonucleotic acid
LytA: Autolysin
PCR: Polymerase chain reaction

## References

[1] Müller BT, Wegener OR, Grabitz K, Pillny M, Thomas L, Sandmann W (2001). Mycotic aneurysms of the thoracic and abdominal aorta and iliac arteries: experience with anatomic and extra-anatomic repair in 33 cases. J Vasc Surg.

[2] Hsu RB, Chen RJ, Wang SS, Chu SH (2004). Infected aortic aneurysms: clinical outcome and risk factor analysis. J Vasc Surg.

[3] Abdeldaim G, Herrmann B, Mölling P, Holmberg H, Blomberg J, Olcén P (2010). Usefulness of real-time PCR for lytA, ply, and Spn9802 on plasma samples for the diagnosis of pneumococcal pneumonia. Clin Microbiol Infect.

[4] Strålin K, Herrmann B, Abdeldaim G, Olcén P, Holmberg H, Mölling P (2014). Comparison of sputum and nasopharyngeal aspirate samples and of the PCR gene targets lytA and Spn9802 for quantitative PCR for rapid detection of pneumococcal pneumonia. J Clin Microbiol.

[5] Carvalho MGS, Tondella ML, McCaustland K, Weidlich L, McGee L, Mayer LW (2007). Evaluation and improvement of real-time PCR assays targeting lytA, ply, and psaA genes for detection of pneumococcal DNA. J Clin Microbiol.

[6] Greve T, Møllert JK (2012). Accurcy of using the lytA gene to distinguish Streptococcus pneumonia from related species. J Med Microbiol..

[7] Macedo TA, Stanson AW, Oderich GS, Johnson CM, Panneton JM, Tie ML (2004). Infected aortic aneurysms: imaging findings. Radiology.

[8] Gornik HL, Creager MA (2008). Aortitis. Circulation.

[9] Luo CY, Ko WC, Kan CD, Lin PY, Yang YJ (2003). In situ reconstruction of septic aortic pseudoaneurysm due to Salmonella or Streptococcus microbial aortitis: long-term follow-up. J Vasc Surg.

[10] Brouwer RE, van Bockel JH, van Dissel JT (1998). Streptococcus pneumoniae, an emerging pathogen in mycotic aneurysms?. Neth J MED.

[11] Cartery C, Astudillo L, Deelchand A, Mouskovitch G, Sailler L, Bossavy JP (2011). Abdominal infectious aortitis caused by streptococcus pneumoniae: a case report and literature review. Ann Vasc Surg.

[12] Dickinson KJ, Parry DJ, Sandoe JA, Gough MJ (2007). Multiple peripheral pneumococcal mycotic aneurysms without aortic involvement: a unique case confirmed with the novel use of a molecular diagnostic technique. J Vasc Surg.

